# Machine learning approach for noninvasive intracranial pressure estimation using pulsatile cranial expansion waveforms

**DOI:** 10.1038/s41746-025-01463-y

**Published:** 2025-01-26

**Authors:** Gustavo Frigieri, Sérgio Brasil, Danilo Cardim, Marek Czosnyka, Matheus Ferreira, Wellingson S. Paiva, Xiao Hu

**Affiliations:** 1 brain4care, Johns Creek, GA USA; 2 Division of Neurosurgery, Department of Neurology, School of Medicine University of São Paulo, Sao Paulo, Brazil; 3 Brain Physics Laboratory, Division of Neurosurgery, Department of Clinical Neurosciences, University of Cambridge, Cambridge, UK; 4 Institute of Electronic Systems, Warsaw University of Technology, Warsaw, Poland; 5 7D Analytics, Sao Paulo, Brazil; 6 Nell Hodgson Woodruff School of Nursing, Emory University, Atlanta, GA USA; 7 Department of Biomedical Engineering, Georgia Institute of Technology & Emory University, Atlanta, GA USA; 8 Department of Biomedical Informatics, School of Medicine, Emory University, Atlanta, GA USA

**Keywords:** Neurology, Medical research

## Abstract

Noninvasive methods for intracranial pressure (ICP) monitoring have emerged, but none has successfully replaced invasive techniques. This observational study developed and tested a machine learning (ML) model to estimate ICP using waveforms from a cranial extensometer device (brain4care [B4C] System). The model explored multiple waveform parameters to optimize mean ICP estimation. Data from 112 neurocritical patients with acute brain injuries were used, with 92 patients randomly assigned to training and testing, and 20 reserved for independent validation. The ML model achieved a mean absolute error of 3.00 mmHg, with a 95% confidence interval within ±7.5 mmHg. Approximately 72% of estimates from the validation sample were within 0-4 mmHg of invasive ICP values. This proof-of-concept study demonstrates that noninvasive ICP estimation via the B4C System and ML is feasible. Prospective studies are needed to validate the model’s clinical utility across diverse settings.

## Background

Research over the past two centuries has shown that maintaining a balance among intracranial compartment volumes is crucial for cerebral function, as elevated intracranial pressure (ICP) reduces cerebral blood perfusion and disrupts cerebral blood flow regulation^1^. The relationship between intracranial volume and pressure, serving as a proxy for intracranial compliance (ICC)^2^, stands as a vital indicator of the state of intracranial dynamics.

In addition to monitoring factors like brain tissue oxygenation, metabolism, and electrical activity, assessing ICC holds paramount importance in the field of neurocritical care^3^. Nevertheless, the gold standard for ICP monitoring, as well as previously proposed ICC monitoring systems, involve placing an invasive probe into the ventricle or brain parenchyma^4,5^. The invasive method, while effective, is limited by the need for specialized personnel to perform the procedure, the substantial cost, and the inherent risks associated with invasive brain procedures.

To address the broader context of elevated ICP, it is essential to recognize the diverse underlying causes contributing to this condition, including traumatic brain injury (TBI), intracranial hemorrhage, hydrocephalus, tumors, and severe ischemic stroke. These conditions disrupt the delicate balance among intracranial compartments, leading to increased ICP and associated complications, such as reduced cerebral perfusion and potential herniation. Identifying populations that could benefit from noninvasive ICP measurement is equally crucial. High-risk groups, such as patients in neurocritical care units, individuals with chronic neurological disorders, or those requiring immediate ICP assessments, such as in the battlefield or emergency settings, would significantly benefit from a safer, noninvasive alternative. The primary advantages of noninvasive methods lie in their potential to reduce procedural risks, enhance accessibility to ICP monitoring in resource-limited settings, and provide dynamic, real-time assessments.

In the pursuit of safer medical practices, modern noninvasive techniques have emerged, offering physicians valuable tools to investigate intracranial hypertension (IH) across various clinical scenarios. These techniques include transcranial Doppler (TCD), optic nerve sheath diameter (ONSD) ultrasonography, pupillometry, etc. Extensive research has been conducted to understand their advantages and limitations, with the consensus indicating satisfactory negative predictive value but low positive predictive power to identify elevated ICP^6^.

TCD was proposed as a method to monitor ICC via a computational approach that takes into account the compartmental compliances of the cerebral arterial bed and the cerebrospinal space. However, direct ICP monitoring could not be replaced with this approach^7^. More recently, an additional noninvasive method has joined this array of tools for investigating ICC and IH—the brain4care (B4C) System^8^. This novel mechanical sensor can detect micrometric pulsatile cranial expansions originated from ICP variations within each cardiac cycle. The B4C System has demonstrated the ability to capture surrogate ICP pulse morphology (ICP waveform—ICPW), a physiological “vital sign” closely associated with ICC^9^. The high sensitivity of the B4C System allows it to detect brain pulsations with amplitudes ranging from 0.04 to 0.80 mm^10^, which result in corresponding micrometric pulsatile cranial expansions. The system also offers automated waveform analysis and translates ICP variations into numeric parameters (such as P2/P1 ratio, time-to-peak [TTP]) in real-time at the bedside, enhancing the dynamic monitoring of patients^11,12^. The P2 wave (tidal wave) follows the P1 wave (percussion wave) in the ICP waveform. An elevated P2/P1 ratio indicates reduced ICC, suggesting the brain’s diminished ability to compensate for increased intracranial volume. Additionally, the TTP (duration from the start of the ICP wave to its highest peak) provides information about intracranial system responsiveness: shorter intervals indicate high ICC, while longer intervals indicate low, detrimental ICC^13^.

Advancements in artificial intelligence (AI), particularly in the realm of machine learning (ML), have shown remarkable potential for brain monitoring, whether it be for clustering arterial blood pressure (ABP) or TCD waveforms to estimate ICP^14^ and ICPW^15^ assessments, or even for identifying complications in ICP monitoring, such as ventriculitis^16^.

Despite these promising developments, the delivering of noninvasive bedside ICP estimation remains elusive. Thus, the primary objective of the present study is to leverage a substantial dataset of ICPW recordings obtained with the B4C System and employ ML techniques to estimate ICP values noninvasively in patients with concurrent invasive ICP monitoring in the neurocritical care setting. Secondarily, this model is compared with a previously reported noninvasive method based on TCD to provide a baseline assessment of clinical performance.

## Results

**Fig. 1:**
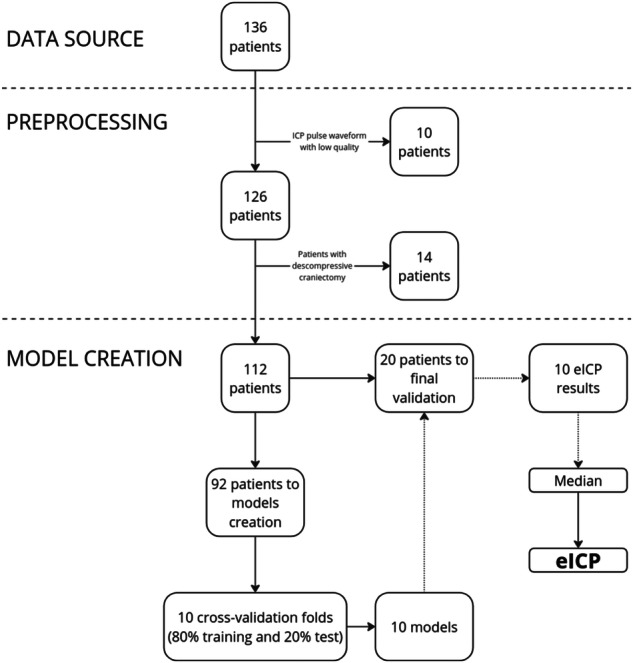
Study design. The figure provides an overview of the data source, patient selection procedures, and model training workflow.

A total of 136 patients were considered for assessment. Ten patients were excluded from the initial set due to the low quality of pooled noninvasive ICPW (nICPW) morphologies (such as the presence of movement artifacts and unreliable waveforms) or the short duration of monitoring, resulting in a revised pool of 126 patients. Subsequently, an additional 14 patients presenting decompressive craniectomy were removed, resulting in 112 patients whose data were segmented into 10-s windows, resulting in a total of 11,604 windows, equivalent to ⁓150,000 pulses (Fig. 1). Table 1 represents the population sample demographics and data allocation for model development and model validation.

**Table 1:** Population demographic characteristics (*n* = 112)

	ICP < 20 mmHg	ICP ≥ 20 mmHg
Sample (10 s-windows), *n* (%)	10,982 (94.64)	622 (5.36)
Age (years) (mean ± SD)	51.60 ± 16.70^a^	43.30 ± 16.40^a^
ICP (mmHg) (mean ± SD)	9.81 ± 4.74^a^	25.10 ± 6.46^a^
CPP (mmHg) (mean ± SD)	80.05 ± 17.04^a^	66.56 ± 14.60^a^
ABP (mmHg) (mean ± SD)	89.85 ± 16.43^a^	91.71 ± 14.90^a^
*Sex (%)*		
Male	76	81
Female	24	19
*Pathology (%)*		
Traumatic brain injury	53	51
Subarachnoid hemorrhage	29	19
Intracerebral hemorrhage	12	23
Ischemic stroke	1	0
Mass	2	1
Subdural hematoma	1	0
Stroke	2	6
Other	0	0
**Center distribution and Data Allocation**	**% of patients (model dataset)**	**% of patients (validation dataset)**
University of Sao Paulo’s Hospital das Clinicas	53.93	68.42
Stanford University	20.22	26.32
Johns Hopkins University	5.62	5.26
University of Porto, Sao Joao Hospital	6.74	0
Federal University of Sao Paulo	5.62	0
Hospital João XXIII	3.93	0
Hospital Estadual de Emergência e Trauma Senador Humberto Lucena	3.93	0

^a^*P* < 0.05, indicating a statistically significant difference between the groups (one-sided Wilcoxon signed-rank test).

Data from 92 patients were preprocessed (9591 10 s windows) and employed to generate and train the ML model. Overall, the entire population sample (*n* = 112) exhibited 5.36% of data with values above 20 mmHg (Table 1). In the model development sample, 86.6% of patients had an external ventricular drain (EVD), and 14.4% of patients had an intraparenchymal ICP transducer, whereas in the validation sample, 100% had an EVD.

ICP was 11.89 ± 3.21 for windows <20 mmHg (*n* = 1996) and 21.26 ± 1.67 for windows >20 mmHg (*n* = 17) in the validation sample. The negative predictive value (NPV) was 0.99 and the positive predictive value (PPV) was 0.14.

**Fig. 2:**
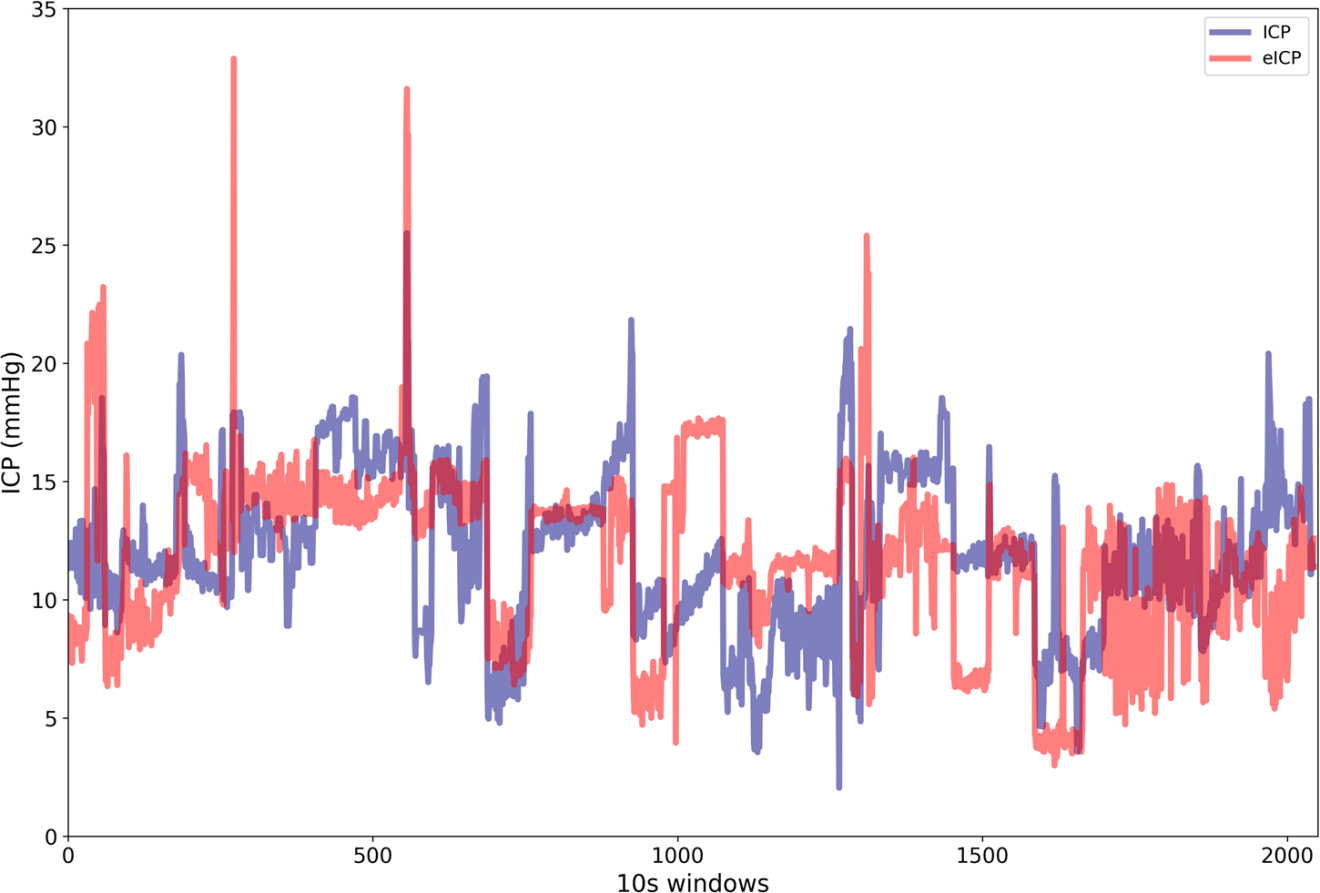
Concurrent trends of invasive and noninvasive intracranial pressure (ICP) methods. The figure displays an overview of 2013 10 s segments of concurrent mean invasive ICP (blue) and noninvasive estimated ICP (eICP) (red) values obtained from the validation dataset (*n* = 20). Individual trends per patient can be observed in Supplementary Figs. 1–3.

The model’s cross-validation folds showed a mean absolute error (MAE) of 1.23 ± 0.02 mmHg and a mean squared error (MSE) of 3.68 ± 0.34 mmHg for the test dataset. For the train dataset, the MAE was 0.91 ± 0.01 mmHg, and the MSE was 1.88 ± 0.05 mmHg. Table 2 shows the model’s cross-validation error distribution for each of the 10 folds. The MAE for the validation dataset was 3.00 mmHg, and the MSE was 13.56 mmHg (*n* = 20, 2013 10-s windows). These values are synchronous with changes in actual ICP, for example as shown in Fig. 2.

**Table 2:** Mean absolute errors (MAE) and mean squared errors (MSE) for the model’s cross-validation folds (values in mmHg)

Fold	Train MAE	Test MAE	Train MSE	Test MSE
1	0.92	1.24	1.95	3.39
2	0.89	1.21	1.82	3.98
3	0.90	1.25	1.81	4.12
4	0.91	1.25	1.83	4.03
5	0.90	1.21	1.83	3.09
6	0.91	1.20	1.88	3.63
7	0.91	1.25	1.93	3.51
8	0.91	1.28	1.90	3.84
9	0.91	1.21	1.90	3.87
10	0.92	1.23	1.91	3.33
Mean ± SD	0.91 ± 0.01	1.23 ± 0.02	1.88 ± 0.05	3.68 ± 0.34

Table 3 shows the noninvasive estimated ICP (eICP) values for the validation dataset individually, which displays the average values of ICP and eICP, along with the observed difference for each patient. A graphical analysis of ICP and eICP trends in time for each validation patient is presented in Supplementary Figs. 1–3.

**Table 3:** Differences between mean ICP and eICP for each patient belonging to the sample of 20 patients set aside for the final replication and validation of the model (values in mmHg)

Patient	MAE	ICP	eICP	eICP-ICP
1	4.27	11.4	11.85	0.45
2	2.06	11.62	9.74	−1.88
3	3.76	14.72	10.96	−3.76
4	4.55	18.25	13.7	−4.55
5	3.5	11.32	14.18	2.86
6	3.55	17.66	16.44	−1.22
7	2.36	14.34	14.58	0.24
8	2.11	7.8	7.95	0.15
9	1.92	13.06	11.77	−1.29
10	4.18	8.68	12.83	4.15
11	4.06	18.23	15.32	−2.91
12	2.39	7.52	7.07	−0.45
13	5.77	11.85	13.47	1.62
14	3.22	15.73	12.53	−3.2
15	3.79	11.86	8.26	−3.6
16	0.79	12.02	11.73	−0.29
17	3.52	7.66	6.5	−1.16
18	2.42	11.07	10.18	−0.89
19	4.79	14.45	9.73	−4.72
20	3.4	14.13	10.85	−3.28

*ICP* intracranial pressure (measured), *eICP* estimated intracranial pressure (noninvasive), *MAE* mean absolute error.

**Fig. 3:**
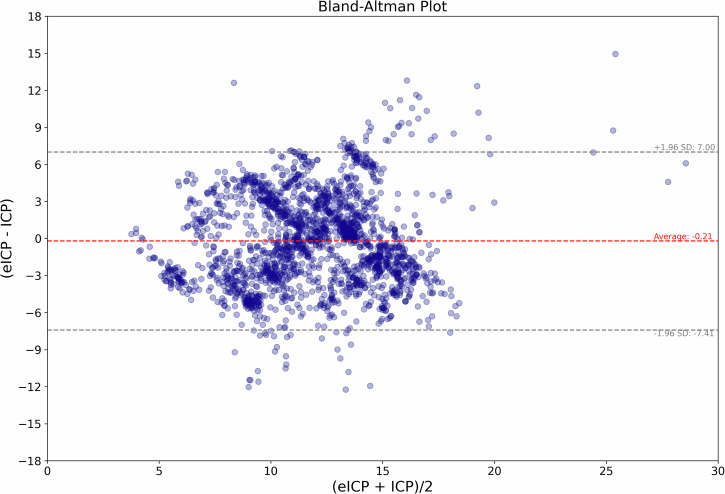
Agreement between invasive and noninvasive intracranial pressure (ICP) methods. The figure represents a Bland–Altman plot comparing actual ICP versus noninvasive estimated ICP (eICP).

**Fig. 4:**
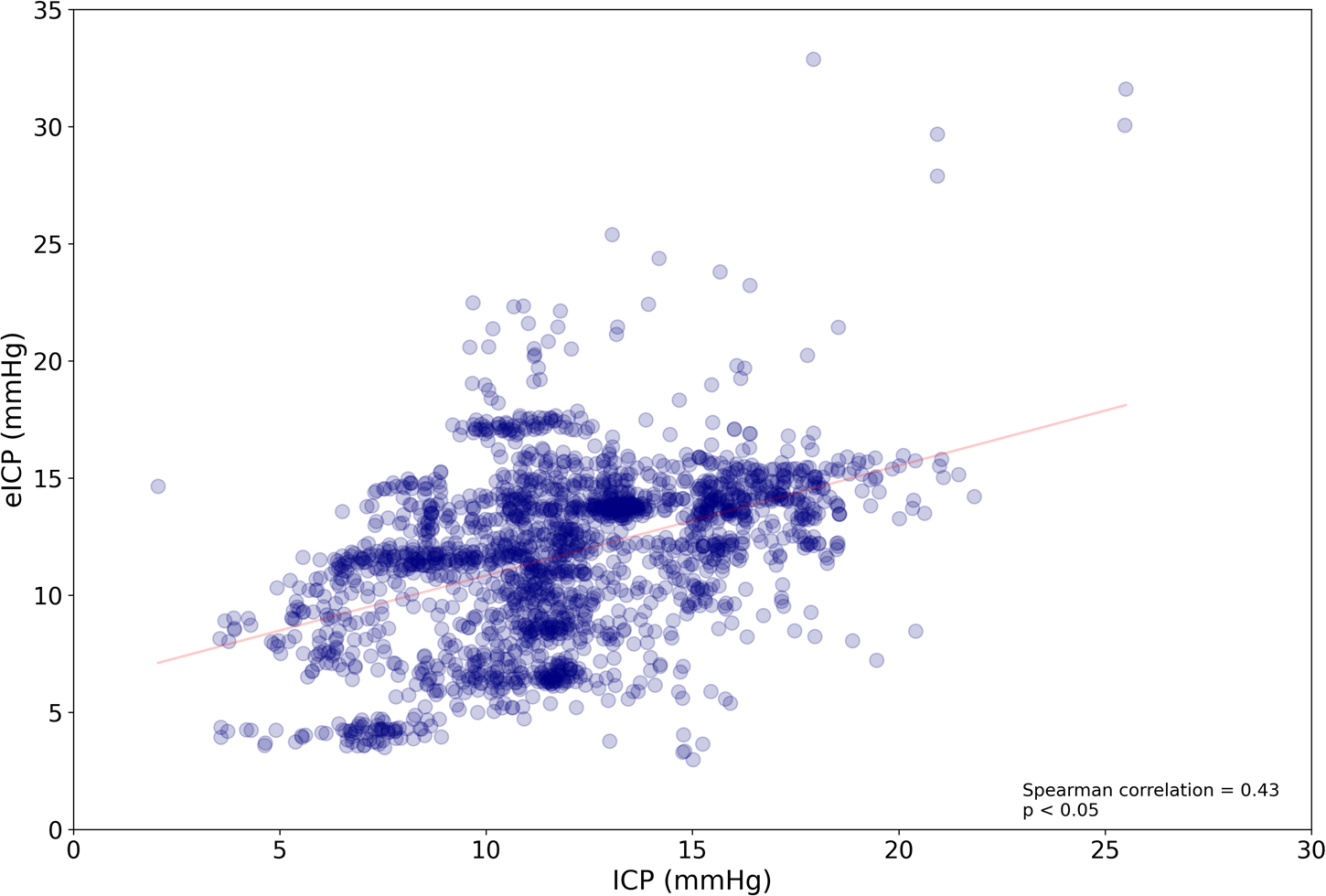
Correlation between invasive and noninvasive intracranial pressure (ICP) methods. Scatterplot of the relationship between actual ICP and noninvasive estimated ICP (eICP). *R* represents the Spearman correlation coefficient.

The Bland–Altman analysis revealed a mean difference (bias) between ICP and eICP of −0.21 mmHg and an SD of ±3.68 mmHg. From these values, a 95% confidence limit for ICP prediction of less than ±7.5 mmHg was achieved (Fig. 3). There was a moderate relationship between ICP and eICP (*r* = 0.43, *p* < 0.05), as shown in Fig. 4.

**Fig. 5:**
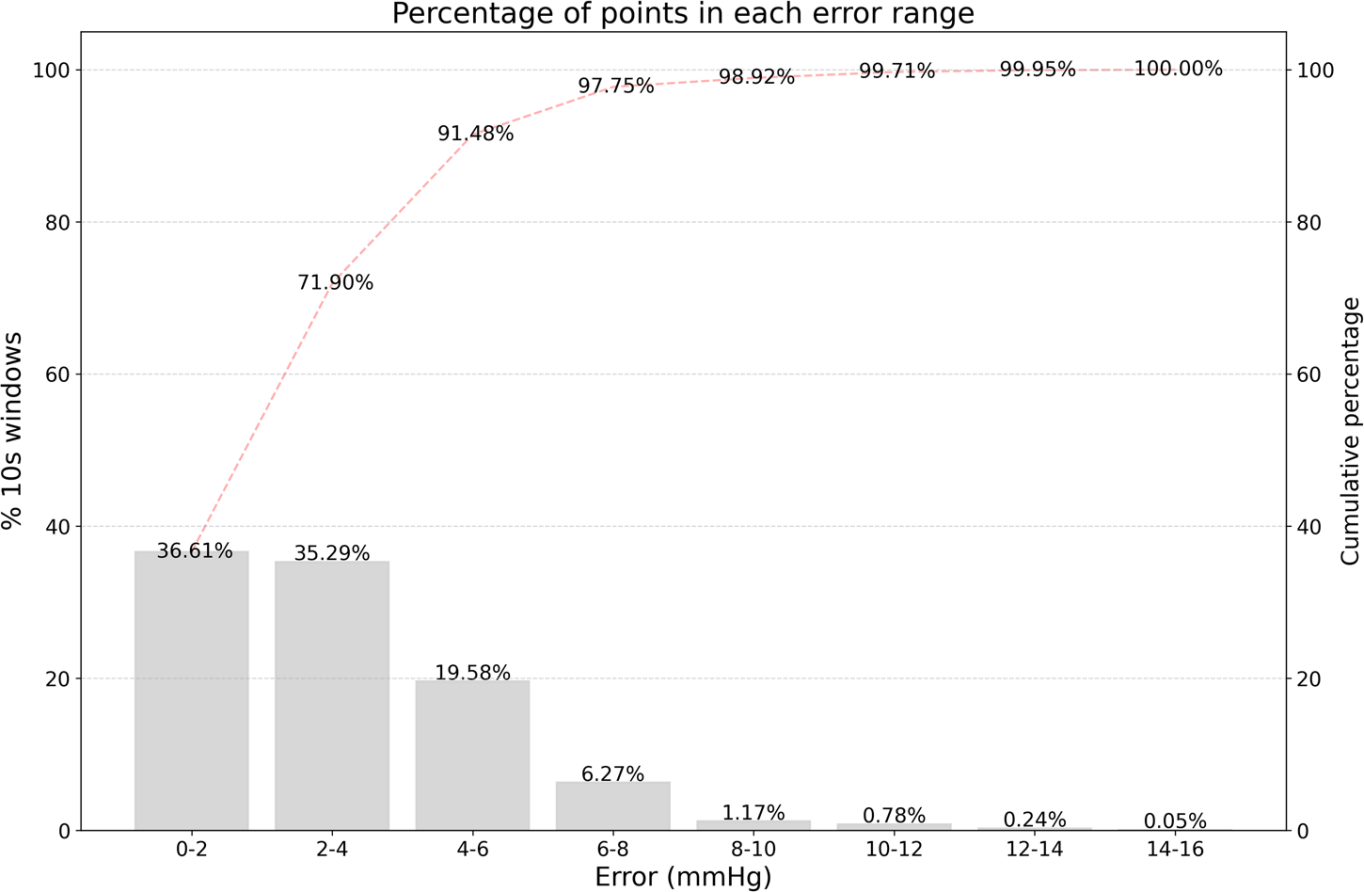
Error distribution histogram for noninvasive intracranial pressure estimation. The figure shows the difference between ICP and eICP values for each 10 s window, showing that <9% of windows showed errors >6 mmHg. ICP intracranial pressure, eICP estimated intracranial pressure.

When computing the eICP means for each 10 s window, differences to actual ICP lower than 2 mmHg were attained in 36.61% of the validation windows, as illustrated in Fig. 5. For 71.90% of the 10 s windows, the difference was below 4 mmHg, and for 91.48%, the error was below 6 mmHg. It is important to note that <9% of the windows exhibited a difference between ICP and eICP >6 mmHg, as observed in Fig. 5. Additionally, an analysis was conducted on the mean difference between the 10 s windows with ICP < 20 mmHg and ICP ≥ 20 mmHg. It was observed that, for the first group, the MAE was 2.97 mmHg, while for the second group, it was 6.41 mmHg. The observed result was expected due to the limited number of data points in the elevated ICP range. Despite efforts to balance the training and testing datasets, 5.36% of data points were above 20 mmHg, limiting effective model training for IH.

### Comparison between eICP and eICP_TCD_

Table 4 shows the eICP and eICP_TCD_ individual values for the validation dataset patients who had concurrent B4C and TCD measurements (*n* = 13), which displays the average values of ICP, eICP, and eICP_TCD_, along with the observed difference for each patient.

**Table 4:** Performance comparison between eICP (brain4care-based) and eICP_TCD_ (transcranial Doppler-based) in a subset of patients in the validation dataset with concurrent brain4care and TCD monitoring (*n* = 13) (values in mmHg)

Patient	ICP	eICP	eICP_TCD_	eICP error	eICP_TCD_ error
1	11.40	11.85	−8.83	0.45	−20.23
2	11.62	9.74	−2.67	−1.88	−14.29
3	14.72	10.96	−10.04	−3.76	−24.76
5	11.32	14.18	−6.67	2.86	−17.99
6	17.66	16.44	−8.65	−1.22	−26.31
11	18.23	15.32	−7.28	−2.91	−25.51
12	7.52	7.07	−7.15	−0.45	−14.67
14	15.73	12.53	−7.40	−3.20	−23.13
16	12.02	11.73	−2.03	−0.29	−14.05
17	7.66	6.50	−3.36	−1.16	−11.02
18	11.07	10.18	−1.03	−0.89	−12.10
19	14.45	9.73	2.63	−4.72	−11.82
20	14.13	10.85	−3.04	−3.28	−17.17

*ICP* intracranial pressure (measured).

The Bland–Altman analysis revealed a bias between ICP and eICP of 1.86 mmHg and an SD of ±2.13 and 18.01 mmHg and an SD of ±5.50 mmHg for ICP and eICP_TCD_. The 95% confidence interval (95% CI) for ICP prediction was ±4.17 mmHg for eICP and ±10.78 mmHg for eICP_TCD_. There was a strong relationship between ICP and eICP (Spearman *r* = 0.76, *p* < 0.05), and no significant relationship between ICP and eICP_TCD_ (Spearman *r* = −0.25, *p* = 0.40).

## Discussion

The proposed ML model, using numerous nICPW features from a large clinical database, estimated mean ICP in 20 acute brain injury (ABI) patients with an MAE of 3.00 mmHg in 2013 validation windows and a 95% CI for ICP prediction below ±7.5 mmHg. These findings advance ICP research and clinical use, promising a reliable noninvasive ICP method applicable beyond neurocritical patients.

The current standards for managing neurocritical patients are undergoing scrutiny, especially when it comes to defining reliable thresholds that indicate when ICP and cerebral perfusion pressure (CPP) values necessitate escalated interventions^17^. Scholars in this field strongly recommend a proactive approach, emphasizing the importance of anticipating IH events to enhance patient outcomes^18,19^. In this context, the role of ICPW becomes pivotal, as changes in these waveforms can serve as early indicators of impending IH surges and are reliable markers of ICC impairment^13^. Additionally, ICPW has been shown to predict patient outcomes effectively^20,21^. With the development of eICP using the practical B4C System, which already processes ICPW parameters like the P2/P1 ratio and TTP, the aim is to make a significant leap in predicting IH events. This development provides a valuable guide in the form of eICP, along with the fine-tuning of predictions through nICPW parameters, thereby offering a comprehensive approach to managing ICP and potentially improving patient outcomes.

To date, various approaches for estimating noninvasive ICP values in humans have been explored. These approaches have employed techniques such as TCD and ABP waveforms^15,22–25^, acoustic signals^26,27^, or ONSD ultrasonography^28^, all showing promising results. However, the literature demonstrates significant variability in the metrics used to determine the accuracy of these noninvasive ICP estimation methods, often measured as the mean absolute difference (MAD), MAE, and 95% CI. In this study, both MAE and MSE were used as evaluation metrics to provide complementary insights into model performance. MAE was selected for its alignment with clinical practice, where average deviations from true values are more intuitively understood and consistent with daily ICP evaluations. MSE was included to emphasize larger deviations, which are particularly critical in ICP measurements due to their potential clinical significance. Furthermore, the use of MAE aligns with regulatory standards, such as those set by the Association for Advancement of Medical Instrumentation (AAMI, NS28:1988 (R2015)) and the Brain Trauma Foundation (BTF) guidelines^29^, which specify accuracy requirements for ICP monitoring.

In one seminal study, Schmidt et al.^24^ proposed a linear model utilizing ABP and TCD cerebral blood velocity to compute noninvasive ICP, reporting a MAD of 4.0 ± 1.8 mmHg. Generally, noninvasive ICP methods within the TCD-based model category have shown varying accuracies, with 95% CI ranging from approximately ±10–20 mmHg^6,14,25,30^. Studies focusing on optic ONSD-based ICP estimation at best presented a 95% CI of ±7.58 mmHg^28^.

A comparative analysis of the model proposed in this study (eICP) with eICP_TCD_, a well-established method in the literature derived from TCD-based CPP^30–32^, highlights notable differences in their performance. While eICP demonstrated a closer agreement with ICP and a stronger correlation, eICP_TCD_ showed less precision (95% CI of ±10.78 mmHg) and no significant relationship with ICP. This comparative analysis within a subset of patients provides a baseline for evaluation and highlights the potential advantages of the proposed model as a more accurate and reliable noninvasive approach. However, a broader validation is warranted, including prospective comparisons across diverse clinical conditions and additional noninvasive ICP estimation methods.

Recent investigations have explored other innovative models for noninvasive ICP estimation. For instance, Jaishankar et al.^22^ developed a frequency-domain model correlating ABP and cerebral blood velocity waveforms. Their study, involving data from five patients, yielded a mean error of −1.5 mmHg and a standard deviation of the error of 4.3 mmHg^22^. Similarly, Megjhani et al.^15^ constructed an ML framework incorporating data from ABP, electrocardiogram (ECG), and TCD waveforms. Their findings indicated an MAE of 3.88 mmHg for the domain adversarial neural network model and 3.94 mmHg for the domain adversarial transformers model, based on data from 13 patients^15^. The authors also indicated a 95% CI in the range of ±10 mmHg^15^.

In another approach, Ganslandt et al.^26^ employed acoustic signal emission techniques, observing a 95% CI of ±7.92 mmHg and a difference of 5 mmHg in 85% of 2543 data points (*n* = 14 patients). More recently, a study using long-distance NIRS data reported that a Random Forest model achieved the lowest MAE of 5.030 mmHg, with a wide 95% CI ranging from 8.782 to −8.487 mmHg^33^. Altogether, these studies highlight the large variations in the accuracy of estimating ICP as well as in the definitions of accuracy metrics to compare invasive and noninvasive ICP.

While clinical guidelines commonly recommend specific threshold values, such as maintaining ICP levels below 22 mmHg and CPP above 60 mmHg to trigger therapy escalation^34^, there is growing recognition that ICP management should be more individualized, and nuanced^35^. This underscores the potential utility of noninvasive ICP estimation as a valuable tool for providing personalized and dynamic ICP management, especially with models capable of high accuracy.

Indeed, the accuracy of ICP measurements is of paramount importance in clinical practice. As per the standards established by the AAMI (NS28:1988 (R2015)) and the BTF guidelines^29^, ICP monitoring devices should have continuous output in the 0–100 mmHg range, with an accuracy of ±2 mmHg in the 0–20 mmHg range, and maximum prediction error of 10% for ICP > 20 mmHg. It is noteworthy that previously proposed noninvasive ICP data-driven models, while valuable, can exhibit varying degrees of mean error, ranging from ±5 to 20 mmHg, often accompanied by high standard deviations^6^.

In neurocritical care, a noninvasive method for real-time ICP estimation is essential for proactive patient management and early intervention, ultimately leading to improved outcomes. While the standard reference values for ICP typically range from 5 to 15 mmHg, there is ongoing debate in the literature regarding the precise thresholds for escalating or de-escalating therapy intensity^17,36,37^. A recent study explored the synergistic relationship between ICP values and the P2/P1 ratio measured with B4C in ABI patients^11^. This study demonstrated that a noninvasive marker of ICC could discriminate short-term outcomes, showing that patients with elevated P2/P1 ratios had poorer outcomes compared to those with normal P2/P1 ratios, even when ICP values were similar between groups (15–25 mmHg).

Therefore, a dynamic and personalized ICP baseline can be derived by continuously monitoring a patient’s intracranial dynamics from admission throughout their clinical course. This aligns with the evolving understanding of intracranial physiology, as emphasized by Godoy et al.^38^, which prioritizes ICC and its impairment—reflected in changes to ICP waveform morphology—over rigid ICP thresholds. For instance, waveform characteristics such as the P2/P1 ratio are sensitive markers of ICC impairment, providing insights into deviations from a patient-specific baseline.

The ML model developed in this study, leveraging brain4care technology, facilitates this personalized approach by delivering noninvasive, real-time ICP estimates based on waveform morphology. Features like the P2/P1 ratio, TTP, and area under the curve enable early detection of ICC impairment, complementing strategies like the “NeuroVanguard” approach^39^, which integrates invasive and noninvasive methods for more accurate assessments. Additionally, adjunct techniques such as TCD, near-infrared spectroscopy (NIRS), and ONSD ultrasound can contribute to baseline determination, offering a multimodal perspective on cerebral compliance, oxygenation, and blood dynamics.

By combining these advanced tools with standard-of-care measures, including neuroimaging and neurological evaluations, clinicians can construct a comprehensive and dynamic baseline tailored to each patient. This personalized approach allows for the early identification of critical trends, enabling timely interventions and reducing reliance on invasive procedures when feasible. Such strategies underscore the value of real-time, noninvasive ICP monitoring and the integration of ML in optimizing the management of intracranial dynamics, ultimately improving neurocritical care outcomes.

The strengths of the B4C System rely on the simplicity of handling, immediate information acquisition, absence of any kind of energy emission, and suitability for lower financial resources locations, as with one device, several patients can be closely monitored^12^. Furthermore, the B4C System’s nICPW represents a true physiological signal of ICP dynamics on a beat-to-beat basis, which is an advantage compared to other models for noninvasive ICP estimation that utilize only secondary parameters related to ICP dynamics, such as TCD-based cerebral blood velocity and mean ABP. In this context, the proposed model is more advantageous because it relies on a single physiological signal, avoiding potential discrepancies arising from a multiparameter architecture. Additionally, for nearly 40% of the validation sample, the proposed model was capable of performing within the error margin recommended by AAMI (±2 mmHg).

The proposed approach, relying on surrogate ICP pulse morphology, simplifies data acquisition and offers better accuracy. Although further refinement is needed, especially for edge cases where the performance of the model is not yet sufficient for standalone clinical decision-making, this study lays the foundation for a clinically viable, noninvasive ICP monitoring tool, with future work focused on dataset expansion and model optimization.

Despite demonstrating superior performance compared to eICP_TCD_ and with previous reports in the field, the proposed ML model requires further prospective validation in larger patient populations to assess its generalizability and robustness across different clinical settings. This will also allow for the collection of a broader range of ICP values, which is limited in the present study. Additionally, the proposed model shows variance based on the training sample composition and is not yet generalizable to clinical conditions outside of ABI.

Patients with large skull defects from procedures like craniectomies can exhibit altered intracranial dynamics, potentially affecting the accuracy of the B4C System^40^. However, the value of ICP pulse morphology in these patients suggests that the B4C System may still provide valuable insights^41^. One notable limitation is the susceptibility to patient agitation, which can lower signal-to-noise ratio (SNR) and hinder waveform readings. Minimizing the impact of patient agitation is crucial for optimizing the system’s performance. A supervised monitoring is suggested to ensure reliable data collection. Another limitation involves the risk of signal contamination from extracranial circulation pulsations if the sensor is mispositioned near major extracranial arteries. Proper sensor positioning can eliminate these artifacts. The B4C System includes control measures, such as an app algorithm that identifies and warns about inadequate signals caused by improper placement or excessive artifacts, ensuring proper data collection for clinical interpretation.

One potential limitation of this study is the utilization of intraparenchymal and intraventricular ICP sensors, which may introduce measurement errors. A systematic review and meta-analysis found these errors between different invasive ICP monitoring methods to be around 1.5 mmHg (95% CI 0.7–2.3)^42^. Although generally negligible, this variability could affect the accuracy of the proposed model, highlighting the need for its careful validation.

The large population sample size used to generate the ML model is a strength of this study compared to previous reports. However, the low diversity of ICP values in the dataset may have contributed to eICP overestimation of actual ICP, even in the 15–20 mmHg range (Fig. 4) which could lead to unnecessary treatment. This indicates that the current approach needs improvement. Future efforts will focus on data augmentation and training the model with larger, more clinically diverse datasets with a higher distribution of IH events. At this stage, the model accuracy is higher when evaluating the average pressure values. Future iterations of the model, prior to seeking clinical regulatory clearance, will be refined using a larger dataset to enhance the estimation dynamics and reduce the temporal fluctuations between underestimation and overestimation.

In summary, the present study demonstrated the potential of estimating ICP using parameters derived exclusively from noninvasively acquired pulsatile cranial expansion waveforms. While further prospective validations are needed to confirm the clinical utility of this ML model, the development of point-of-care noninvasive ICP estimation shows promise. This advancement could make ICP a more widely accessible vital sign, potentially improving the identification, treatment selection, and prognostication processes in various clinical contexts.

## Methods

### Study design and population

This is a retrospective analysis of data from prospective observational studies conducted independently at four centers in Brazil, including the Sao Paulo University’s Hospital das Clinicas (IRB under the reference number 6150621, CAAE: 39348920.1.1001.0068), the Federal University of Sao Paulo (IRB reference number 3.129.120, CAAE: 03843118.0.0000.5505), Hospital João XXIII (IRB under the reference number 6150621, CAAE: 39348920.1.1001.0068) and Hospital Estadual de Emergência e Trauma Senador Humberto Lucena (IRB reference number 5.078.425, CAAE: 39348920.1.2001.5186); one center in Portugal, the University of Porto’s Sao Joao Hospital (IRB reference number 106-17); and two centers in the United States, Stanford University (IRB reference number 46100) and Johns Hopkins University (IRB reference number IRB00204065). The study protocol was conducted in accordance with the Declaration of Helsinki and received approval from the local Ethics Committee in each respective center, and informed consent was obtained from the patients or their legally authorized representatives. This study was performed according to the Strengthening the Reporting of Observational Studies in Epidemiology (STROBE) standards (https://www.strobe-statement.org/checklists/).

Patients admitted at each respective center in the period of 1 January 2015 to 1 January 2024, were eligible for inclusion in this study if they had experienced an ABI requiring invasive ICP monitoring within the first five days of their hospital admission. Patients who had undergone decompressive craniectomy or exhibited signs of brain death were excluded from the study. For cases involving TBI, the guidelines for the high risk of brain herniation established by the BTF guidelines^29^ were followed. In the case of subarachnoid hemorrhage, management procedures were similar, although it is important to note that specific guidelines for ICP management in non-traumatic ABI cases are currently lacking^43^. It is worth mentioning that patient care was conducted independently of this study, and the data obtained using the B4C sensor was not utilized for clinical decision-making or patient management.

### Neuromonitoring

ICP was monitored via an optic-fiber intraparenchymal transducer (Raumedic, Munchberg, Germany) or through an EVD levelled at the height of the external auditory meatus, according to clinical indications. The B4C System (brain4care, Sao Carlos, Sao Paulo, Brazil), a wearable sensor cleared by the Food and Drug Administration (FDA number K201989), was used to register nICPW. Previous studies described its principle of operation in detail^8^. In summary, the B4C System involves placing a highly sensitive sensor in contact with the skin over the skull which detects micrometric pulsatile cranial expansions originating from ICP variations each cardiac cycle. Morphological features from the waveforms obtained using the B4C System have been consistently demonstrated to correlate with invasive ICP in multiple clinical studies^11,12,40,44,45^.

The B4C System was placed in the frontotemporal region on the same side as the ICP probe implantation. Simultaneous recordings taken from distinct patient samples were made of invasive ABP, ICP, and noninvasive pulsatile cranial expansion waveforms (B4C System). For the data collected at the Sao Paulo University’s Hospital das Clinicas, additional recordings of ECG, temperature, oxygen saturation, and middle cerebral artery (MCA) blood velocity with TCD were taken. The primary purpose of establishing this database was to investigate variations in ICP in relation to MCA blood velocities and cerebrovascular autoregulation^46,47^, as well as invasive and noninvasive ICP waveforms^40^.

The collected data consisted of several short recording sessions, each lasting at least 10 min, and electronically acquired and synchronized from patient bedside monitors at a sampling frequency of 250 Hz using an in-house data collection system (brain4care, Sao Carlos, Sao Paulo, Brazil). During these sessions, strict monitoring by the investigator in charge was maintained to prevent any displacement of the B4C sensor, which could potentially compromise the quality of the signal. Investigators participating in the data collection were not part of the direct care team. Also, the direct care team did not have access to the data during collection.

### Signal processing

Initially, the power spectral density derived from the B4C signal was used to estimate the SNR, calculated as the ratio of signal energy within the fundamental frequency and its first three harmonic frequencies, compared to the spectral range spanning from 0.1 to 25 Hz. Signals exceeding an SNR threshold of 0.35 underwent mean pulse assessment serving as the input for a quality classifier to determine signal morphology acceptability. The output of this classifier indicated whether a signal met quality standards, having passed these two assessment stages. For downsampled signals, standardizing sample rates ensured uniformity across all signals. The data curation process was rigorous, with initial data parsing, detrending, signal validation, filtering, inversion verification, pulse identification, artifact removal, pulse alignment, pulse averaging, and pulse parameter calculation performed by analytical software in the B4C System cloud. Signal filtering involved a two-part algorithm: a 0–1 signal quality index based on spectral analysis of one-minute downsampled and detrended data, followed by a binary classifier trained on 21,000 manually classified pulses.

For the invasive ICP signal, no signal processing was applied to derive the averaged ICP values.

### Proposed machine learning framework

The model utilized for estimating ICP employed a histogram gradient boosting (HGB) regressor^48^, an ensemble learning technique that sequentially trains decision trees to correct errors made by previous trees. This approach effectively captures complex relationships and non-linear patterns in the data by constructing trees based on histograms of feature values. HGB was the model of choice based on a comparative analysis with other models such as temporal convolutional network (TCN) and convolutional neural network (CNN), as it provided the best estimation accuracy and requires less computational power. A quantitative comparison of the performance of each model tested is presented in Supplementary Table 1.

The feature engineering process involved extracting various parameters from the B4C waveform. This process resulted in the extraction of approximately 1110 features, described in Supplementary Note 1. These features were then refined based on their Spearman correlation coefficient with the regression target—the invasively obtained ICP values in these patients, and subsequently with their calculated importance. Feature importance was determined by prioritizing features with higher correlation coefficients. Redundant features were excluded in favor of the next most important feature, resulting in 15 features. The correlation coefficients between each feature and ICP are also described in Supplementary Note 1. The detection of redundant features was based on two types of correlation. First, the correlation between features is assessed. If two features are highly correlated, it indicates they provide similar information, which can lead to redundancy. Redundant features are excluded in favor of the next most important feature, ensuring that only unique and valuable information is retained. This step helps eliminate unnecessary complexity without sacrificing predictive power. Second, the correlation between each feature and the target variable is evaluated. Features that show little to no correlation with the target are unlikely to contribute meaningfully to the model’s predictions and are subsequently excluded.

To optimize model performance, hyperparameters underwent selection through a grid search approach with a primary focus on achieving the optimal MAE and MSE for the training dataset while avoiding undersampling or oversampling through cross-validation with 10 folds and analysis of hyperparameters. Table 5 describes the key elements for processing and optimization of the proposed model. Supplementary Table 2 summarizes results obtained for each combination of hyperparameters to arrive at the final model selection.

**Table 5:** Key elements for processing and optimization of the proposed model

Objective	Method	Metric for evaluation, validation and optimization	Hyperparameters	Optimization strategy	Generalization technique	Generalization Hyperparameters
Regression	Histogram gradient boosting	MAE and MSE	4000 trees; max tree depth = 15; regularization = 0.5	Tune number of trees and depth using grid-search method	Bootstrap	max number of leaf nodes = 50; min number of samples per leaf = 80; learning rate = 0.005; number of histogram bins = 255

*MAE* mean absolute error, *MSE* mean squared error, *max* maximum, *min* minimum.

To address the imbalances in the dataset, e.g., the limited representation of IH data (ICP > 20 mmHg), a class weighting scheme that assigns weights inversely proportional to the frequency of each class was implemented for the model development phase. This method ensures that cases with lower representativity, e.g., IH events, are given greater importance during the training process, thereby improving the model’s performance on these critical cases. By calculating the frequency of normal and elevated ICP instances and mapping these frequencies to their corresponding weights, the model’s ability to learn from the imbalanced data can be enhanced, ultimately leading to more accurate predictions across all ICP ranges.

Parameters derived from B4C nICPW morphological segments (e.g., P2/P1 ratio, TTP, area under the curve, intracranial compliance scale^49^) and outcomes of dimensionality reduction techniques were utilized in the development of this proprietary (patent-pending) predictive model. The Isomap dimensionality reduction technique was applied to the raw waveform signals, resulting in 25 reduced-dimensional fragments (Supplementary Note 1) used as input features for the model. To ensure model simplicity and avoid redundancy, the inclusion of these fragments was carefully analyzed alongside the raw features. Features were refined based on their correlation with the regression target (ICP) and their importance to the model as described above. Importantly, no parameters derived from any other physiological signals were included in the model.

The entire workflow embraced a robust 10-fold cross-validation strategy to ensure the reliability of the model. This strategy involved separating data from the same patient between training and testing in each fold, with 80% allocated for model training and 20% for testing. Additionally, data from 20 patients were set aside for final replication and validation, with their data held separate and not utilized in the model development phase.

After model training, saved models from each fold were used to estimate ICP values in the validation dataset (*n* = 20). The model produced 10 calibrations and the resulting estimated ICP value (eICP) was determined as the median of these calibration models.

Python 3.7, in a Jupyter Notebook environment hosted on a high-performance server, was utilized for model development due to its flexibility and extensive libraries for ML tasks.

### Comparison with a baseline noninvasive method

Czosnyka et al.^50^ proposed a method based on diastolic cerebral blood velocity for the estimation of noninvasive cerebral perfusion pressure (nCPP). Studies in the literature have demonstrated that such patterns of the TCD waveform reflect impaired cerebral perfusion caused by a decrease in CPP^50,51^. With this method, ICP can be estimated as the difference between ABP and nCPP (eICP_TCD_ = ABP−nCPP)^30–32^. nCPP estimation is expressed as (Eq. (1)):

1 $${\rm{nCPP}}={\rm{ABP}}\times \frac{{\rm{F}}{{\rm{V}}}_{{\rm{d}}}}{{\rm{F}}{{\rm{V}}}_{{\rm{m}}}}+14\,{\rm{mmHg}}$$

FV_d_ and FV_m_ (cm/s) represent diastolic and mean blood velocity from the MCA, respectively. 14 mmHg is a preestablished calibration (zeroing) parameter derived from TBI patients.

To provide a comparative baseline assessment with an established method previously reported in the literature, the proposed eICP model was compared with eICP_TCD_ in a subset of cases from the validation dataset with patients who had concurrent B4C and TCD measurements (*n* = 13).

### Statistical analyses

The model was statistically assessed through the utilization of MAE, MSE, Bland–Altman plot for comparison of ICP with eICP, 95% CI for ICP prediction, and the Spearman correlation between ICP and eICP values. The Shapiro–Wilk test was used to test the normality of the distribution of the variables. Mean ± standard deviation (SD) was utilized to evaluate the outcomes of the patient sample. A *P* value < 0.05 was considered for statistical significance. The statistical calculations were also carried out using Python 3.7.

For this observational study, the sample size was determined based on the availability of participants meeting inclusion criteria and practical constraints such as time and resources. While acknowledging the convenience-based nature of the sample, efforts were made to ensure representativeness within these limitations.

## Supplementary information

Supplementary Materials

## Data Availability

The datasets used or analyzed during the current study are available from the corresponding author on reasonable request.

## Code availability

The underlying code for this study [and training/validation datasets] is not publicly available for proprietary reasons.
