# Supplementary material for: Machine learning approach for noninvasive intracranial pressure estimation using pulsatile cranial expansion waveforms
Source: NPJ Digit Med. 2025 Jan 26;8:57. doi: 10.1038/s41746-025-01463-y (PMC11770073; doi:10.1038/s41746-025-01463-y)

Supplementary Table 1. Quantitative comparison of the performance of all models tested. The table below presents the averaged mean absolute error (MAE, in mmHg) for ICP and eICP per patient belonging to the sample of 20 patients set aside for the final replication and validation of the model. On a 10-s window level (n=2013), the averaged MAEs were 3.00 mmHg, 3.11 mmHg and 3.29 mmHg for HGB (histogram gradient boosting), CNN (convolutional neural network), TCN (temporal convolutional network), respectively.

ID	MAE HGB	MAE TCN	MAE CNN
**1**	4.27	2.39	2.92
**2**	2.06	0.69	1.51
**3**	3.76	1.55	1.66
**4**	4.55	9.44	7.46
**5**	3.50	1.59	1.34
**6**	3.55	5.15	5.90
**7**	2.36	3.02	3.05
**8**	2.11	2.63	2.5
**9**	1.92	2.18	2.67
**10**	4.18	3.84	4.78
**11**	4.06	1.73	3.91
**12**	2.39	7.82	7.27
**13**	5.77	1.89	1.68
**14**	3.22	3.21	2.60
**15**	3.79	6.31	6.13
**16**	0.79	4.05	5.27
**17**	3.52	3.30	3.51
**18**	2.42	2.05	2.16
**19**	4.79	2.94	2.21
**20**	3.40	3.57	3.92

Supplementary Note 1. The features used in the model can be categorized into three distinct groups. The first group consists of features extracted directly from the morphology of the intracranial pressure pulse, including parameters such as amplitude, areas, initial and final angles, derivatives, the P2/P1 ratio, and time-to-peak (TTP) (categories 1, 2, 3, and 4). The second group includes features generated through dimensionality reduction using Isomap, resulting in 25 new features (category 5). The third group comprises interaction features, created by pairwise multiplication of the initially generated features (category 6). These features, together, provide a comprehensive representation of ICP waveform morphology, timing, and dynamics. Their extraction and refinement were guided by their relevance to ICP prediction, determined through statistical correlation and feature importance analysis. The diversity and specificity of these features ensure that the model captures both global trends and subtle nuances in ICP dynamics, offering robust predictive capabilities.

1. Morphological Features

These parameters describe the shape and structure of the ICP waveform:

- P2/P1 Ratio: The ratio between P2 and P1 amplitudes of the ICP waveform, which indicates intracranial compliance impairment when P2 surpasses P1.
- Amp (Pulse Amplitude): The difference between the maximum and minimum values of the waveform, indicating overall pulse strength.

2. Temporal Features

These capture the timing aspects of the waveform:

- TTP (Time-to-Peak): The time interval from the start of the pulse to the highest peak, reflecting waveform timing dynamics.
- Der (Initial Derivative of the Pulse): The slope of the waveform's initial rise, associated with the rapidity of pressure change.

3. Area-Based Features

These parameters analyze the distribution of the waveform area, divided into segments to capture nuanced dynamics:

- At (Total Area): The total area under the waveform curve.
- A1–A5: The proportion of the total area under specific segments of the pulse curve:
- A1: Area from 0–20% of the pulse.
- A2: Area from 20–40% of the pulse.
- A3: Area from 40–60% of the pulse.
- A4: Area from 60–80% of the pulse.
- A5: Area from 80–100% of the pulse.
- Area Ratios (e.g., A2/A1, A3/A1): Ratios between these areas, capturing the relative contribution of different segments to the total area.

4. Proprietary Indices

- ICS (Intracranial Compliance Scale): A proprietary index quantifying intracranial compliance, based on the overall waveform morphology.

5. Dimensionality-Reduced Features

To simplify the dataset and reduce redundancy, the Isomap dimensionality reduction technique was applied, resulting in 25 fragments (I1–I25). These fragments capture complex waveform dynamics in a compact representation.

6. Relational Features

These involve interactions and correlations between key waveform parameters:

- Corr_P2P1_Amp: Pearson correlation between the P2/P1 ratio and pulse amplitude, reflecting their interdependence.

- Corr_TTP_Amp: Pearson correlation between time-to-peak and pulse amplitude, indicating how waveform timing relates to its strength.

A List of all features extracted from the brain4care pulse waveform. **Acronyms:** A (1-5): segmented area under the curve, where one unit represents 20% of the waveform length; Amp: waveform amplitude; At: total area under the curve; Corr: correlation coefficient; Der: waveform slope derivative; I (1-25): ISOMAP dimensionality reduction elements; ICS: intracranial compliance scale; P2/P1: ratio between P1 and P2 ICP waveform peaks; TTP: time-to-peak.

['P2P1', 'TTP', 'ICS', 'Der', 'At', 'A1', 'A2', 'A3', 'A4', 'A5', 'Amp', 'I1', 'I2', 'I3', 'I4', 'I5', 'I6', 'I7', 'I8', 'I9', 'I10', 'I11', 'I12', 'I13', 'I14', 'I15', 'I16', 'I17', 'I18', 'I19', 'I20', 'I21', 'I22', 'I23', 'I24', 'I25', 'A2A1', 'A3A1', 'A4A1', 'A5A1', 'A3A2', 'A4A2', 'A5A2', 'A4A3', 'A5A3', 'A5A4', 'Corr_P2P1_Amp', 'Corr_TTP_Amp', 'P2P1_TTP', 'P2P1_ICS', 'P2P1_Der', 'P2P1_At', 'P2P1_A1', 'P2P1_A2', 'P2P1_A3', 'P2P1_A4', 'P2P1_A5', 'P2P1_Amp', 'P2P1_I1', 'P2P1_I2', 'P2P1_I3', 'P2P1_I4', 'P2P1_I5', 'P2P1_I6', 'P2P1_I7', 'P2P1_I8', 'P2P1_I9', 'P2P1_I10', 'P2P1_I11', 'P2P1_I12', 'P2P1_I13', 'P2P1_I14', 'P2P1_I15', 'P2P1_I16', 'P2P1_I17', 'P2P1_I18', 'P2P1_I19', 'P2P1_I20', 'P2P1_I21', 'P2P1_I22', 'P2P1_I23', 'P2P1_I24', 'P2P1_I25', 'P2P1_Corr_P2P1_Amp', 'P2P1_Corr_TTP_Amp', 'P2P1_A2A1', 'P2P1_A3A1', 'P2P1_A4A1', 'P2P1_A5A1', 'P2P1_A3A2', 'P2P1_A4A2', 'P2P1_A5A2', 'P2P1_A4A3', 'P2P1_A5A3', 'P2P1_A5A4', 'TTP_ICS', 'TTP_Der', 'TTP_At', 'TTP_A1', 'TTP_A2', 'TTP_A3', 'TTP_A4', 'TTP_A5', 'TTP_Amp', 'TTP_I1', 'TTP_I2', 'TTP_I3', 'TTP_I4', 'TTP_I5', 'TTP_I6', 'TTP_I7', 'TTP_I8', 'TTP_I9', 'TTP_I10', 'TTP_I11', 'TTP_I12', 'TTP_I13', 'TTP_I14', 'TTP_I15', 'TTP_I16', 'TTP_I17', 'TTP_I18', 'TTP_I19', 'TTP_I20', 'TTP_I21', 'TTP_I22', 'TTP_I23', 'TTP_I24', 'TTP_I25', 'TTP_Corr_P2P1_Amp', 'TTP_Corr_TTP_Amp', 'TTP_A2A1', 'TTP_A3A1', 'TTP_A4A1', 'TTP_A5A1', 'TTP_A3A2', 'TTP_A4A2', 'TTP_A5A2', 'TTP_A4A3', 'TTP_A5A3', 'TTP_A5A4', 'ICS_Der', 'ICS_At', 'ICS_A1', 'ICS_A2', 'ICS_A3', 'ICS_A4', 'ICS_A5', 'ICS_Amp', 'ICS_I1', 'ICS_I2', 'ICS_I3', 'ICS_I4', 'ICS_I5', 'ICS_I6', 'ICS_I7', 'ICS_I8', 'ICS_I9', 'ICS_I10', 'ICS_I11', 'ICS_I12', 'ICS_I13', 'ICS_I14', 'ICS_I15', 'ICS_I16', 'ICS_I17', 'ICS_I18', 'ICS_I19', 'ICS_I20', 'ICS_I21', 'ICS_I22', 'ICS_I23', 'ICS_I24', 'ICS_I25', 'ICS_Corr_P2P1_Amp', 'ICS_Corr_TTP_Amp', 'ICS_A2A1', 'ICS_A3A1', 'ICS_A4A1', 'ICS_A5A1', 'ICS_A3A2', 'ICS_A4A2', 'ICS_A5A2', 'ICS_A4A3', 'ICS_A5A3', 'ICS_A5A4', 'Der_At', 'Der_A1', 'Der_A2', 'Der_A3', 'Der_A4', 'Der_A5', 'Der_Amp', 'Der_I1', 'Der_I2', 'Der_I3', 'Der_I4', 'Der_I5', 'Der_I6', 'Der_I7', 'Der_I8', 'Der_I9', 'Der_I10', 'Der_I11', 'Der_I12', 'Der_I13', 'Der_I14', 'Der_I15', 'Der_I16', 'Der_I17', 'Der_I18', 'Der_I19', 'Der_I20', 'Der_I21', 'Der_I22', 'Der_I23', 'Der_I24', 'Der_I25', 'Der_Corr_P2P1_Amp', 'Der_Corr_TTP_Amp', 'Der_A2A1', 'Der_A3A1', 'Der_A4A1', 'Der_A5A1', 'Der_A3A2', 'Der_A4A2', 'Der_A5A2', 'Der_A4A3', 'Der_A5A3', 'Der_A5A4', 'At_A1', 'At_A2', 'At_A3', 'At_A4', 'At_A5', 'At_Amp', 'At_I1', 'At_I2', 'At_I3', 'At_I4', 'At_I5', 'At_I6', 'At_I7', 'At_I8', 'At_I9', 'At_I10', 'At_I11', 'At_I12', 'At_I13', 'At_I14', 'At_I15', 'At_I16', 'At_I17', 'At_I18', 'At_I19', 'At_I20', 'At_I21', 'At_I22', 'At_I23', 'At_I24', 'At_I25', 'At_Corr_P2P1_Amp', 'At_Corr_TTP_

Amp', 'At_A2A1', 'At_A3A1', 'At_A4A1', 'At_A5A1', 'At_A3A2', 'At_A4A2', 'At_A5A2', 'At_A4A3', 'At
_A5A3', 'At_A5A4', 'A1_A2', 'A1_A3', 'A1_A4', 'A1_A5', 'A1_Amp', 'A1_I1', 'A1_I2', 'A1_I3', 'A1_I4',
'A1_I5', 'A1_I6', 'A1_I7', 'A1_I8', 'A1_I9', 'A1_I10', 'A1_I11', 'A1_I12', 'A1_I13', 'A1_I14', 'A1_I15', 'A
1_I16', 'A1_I17', 'A1_I18', 'A1_I19', 'A1_I20', 'A1_I21', 'A1_I22', 'A1_I23', 'A1_I24', 'A1_I25', 'A1_Co
rr_P2P1_Amp', 'A1_Corr_TTP_Amp', 'A1_A2A1', 'A1_A3A1', 'A1_A4A1', 'A1_A5A1', 'A1_A3A2', 'A1
_A4A2', 'A1_A5A2', 'A1_A4A3', 'A1_A5A3', 'A1_A5A4', 'A2_A3', 'A2_A4', 'A2_A5', 'A2_Amp', 'A2_I1
', 'A2_I2', 'A2_I3', 'A2_I4', 'A2_I5', 'A2_I6', 'A2_I7', 'A2_I8', 'A2_I9', 'A2_I10', 'A2_I11', 'A2_I12', 'A2_
I13', 'A2_I14', 'A2_I15', 'A2_I16', 'A2_I17', 'A2_I18', 'A2_I19', 'A2_I20', 'A2_I21', 'A2_I22', 'A2_I23',
'A2_I24', 'A2_I25', 'A2_Corr_P2P1_Amp', 'A2_Corr_TTP_Amp', 'A2_A2A1', 'A2_A3A1', 'A2_A4A1', '
A2_A5A1', 'A2_A3A2', 'A2_A4A2', 'A2_A5A2', 'A2_A4A3', 'A2_A5A3', 'A2_A5A4', 'A3_A4', 'A3_A5',
'A3_Amp', 'A3_I1', 'A3_I2', 'A3_I3', 'A3_I4', 'A3_I5', 'A3_I6', 'A3_I7', 'A3_I8', 'A3_I9', 'A3_I10', 'A3_I
11', 'A3_I12', 'A3_I13', 'A3_I14', 'A3_I15', 'A3_I16', 'A3_I17', 'A3_I18', 'A3_I19', 'A3_I20', 'A3_I21', '
A3_I22', 'A3_I23', 'A3_I24', 'A3_I25', 'A3_Corr_P2P1_Amp', 'A3_Corr_TTP_Amp', 'A3_A2A1', 'A3_A
3A1', 'A3_A4A1', 'A3_A5A1', 'A3_A3A2', 'A3_A4A2', 'A3_A5A2', 'A3_A4A3', 'A3_A5A3', 'A3_A5A4',
'A4_A5', 'A4_Amp', 'A4_I1', 'A4_I2', 'A4_I3', 'A4_I4', 'A4_I5', 'A4_I6', 'A4_I7', 'A4_I8', 'A4_I9', 'A4_I1
0', 'A4_I11', 'A4_I12', 'A4_I13', 'A4_I14', 'A4_I15', 'A4_I16', 'A4_I17', 'A4_I18', 'A4_I19', 'A4_I20', 'A
4_I21', 'A4_I22', 'A4_I23', 'A4_I24', 'A4_I25', 'A4_Corr_P2P1_Amp', 'A4_Corr_TTP_Amp', 'A4_A2A1
', 'A4_A3A1', 'A4_A4A1', 'A4_A5A1', 'A4_A3A2', 'A4_A4A2', 'A4_A5A2', 'A4_A4A3', 'A4_A5A3', 'A4_
A5A4', 'A5_Amp', 'A5_I1', 'A5_I2', 'A5_I3', 'A5_I4', 'A5_I5', 'A5_I6', 'A5_I7', 'A5_I8', 'A5_I9', 'A5_I10
', 'A5_I11', 'A5_I12', 'A5_I13', 'A5_I14', 'A5_I15', 'A5_I16', 'A5_I17', 'A5_I18', 'A5_I19', 'A5_I20', 'A5
_I21', 'A5_I22', 'A5_I23', 'A5_I24', 'A5_I25', 'A5_Corr_P2P1_Amp', 'A5_Corr_TTP_Amp', 'A5_A2A1'
, 'A5_A3A1', 'A5_A4A1', 'A5_A5A1', 'A5_A3A2', 'A5_A4A2', 'A5_A5A2', 'A5_A4A3', 'A5_A5A3', 'A5_
A5A4', 'Amp_I1', 'Amp_I2', 'Amp_I3', 'Amp_I4', 'Amp_I5', 'Amp_I6', 'Amp_I7', 'Amp_I8', 'Amp_I9', '
Amp_I10', 'Amp_I11', 'Amp_I12', 'Amp_I13', 'Amp_I14', 'Amp_I15', 'Amp_I16', 'Amp_I17', 'Amp_I1
8', 'Amp_I19', 'Amp_I20', 'Amp_I21', 'Amp_I22', 'Amp_I23', 'Amp_I24', 'Amp_I25', 'Amp_Corr_P2P
1_Amp', 'Amp_Corr_TTP_Amp', 'Amp_A2A1', 'Amp_A3A1', 'Amp_A4A1', 'Amp_A5A1', 'Amp_A3A2
', 'Amp_A4A2', 'Amp_A5A2', 'Amp_A4A3', 'Amp_A5A3', 'Amp_A5A4', 'I1_I2', 'I1_I3', 'I1_I4', 'I1_I5',
'I1_I6', 'I1_I7', 'I1_I8', 'I1_I9', 'I1_I10', 'I1_I11', 'I1_I12', 'I1_I13', 'I1_I14', 'I1_I15', 'I1_I16', 'I1_I17', '
I1_I18', 'I1_I19', 'I1_I20', 'I1_I21', 'I1_I22', 'I1_I23', 'I1_I24', 'I1_I25', 'I1_Corr_P2P1_Amp', 'I1_Corr_
TTP_Amp', 'I1_A2A1', 'I1_A3A1', 'I1_A4A1', 'I1_A5A1', 'I1_A3A2', 'I1_A4A2', 'I1_A5A2', 'I1_A4A3', 'I
1_A5A3', 'I1_A5A4', 'I2_I3', 'I2_I4', 'I2_I5', 'I2_I6', 'I2_I7', 'I2_I8', 'I2_I9', 'I2_I10', 'I2_I11', 'I2_I12', 'I
2_I13', 'I2_I14', 'I2_I15', 'I2_I16', 'I2_I17', 'I2_I18', 'I2_I19', 'I2_I20', 'I2_I21', 'I2_I22', 'I2_I23', 'I2_I2
4', 'I2_I25', 'I2_Corr_P2P1_Amp', 'I2_Corr_TTP_Amp', 'I2_A2A1', 'I2_A3A1', 'I2_A4A1', 'I2_A5A1', 'I
2_A3A2', 'I2_A4A2', 'I2_A5A2', 'I2_A4A3', 'I2_A5A3', 'I2_A5A4', 'I3_I4', 'I3_I5', 'I3_I6', 'I3_I7', 'I3_I8'
, 'I3_I9', 'I3_I10', 'I3_I11', 'I3_I12', 'I3_I13', 'I3_I14', 'I3_I15', 'I3_I16', 'I3_I17', 'I3_I18', 'I3_I19', 'I3_I
20', 'I3_I21', 'I3_I22', 'I3_I23', 'I3_I24', 'I3_I25', 'I3_Corr_P2P1_Amp', 'I3_Corr_TTP_Amp', 'I3_A2A1
', 'I3_A3A1', 'I3_A4A1', 'I3_A5A1', 'I3_A3A2', 'I3_A4A2', 'I3_A5A2', 'I3_A4A3', 'I3_A5A3', 'I3_A5A4',
'I4_I5', 'I4_I6', 'I4_I7', 'I4_I8', 'I4_I9', 'I4_I10', 'I4_I11', 'I4_I12', 'I4_I13', 'I4_I14', 'I4_I15', 'I4_I16', 'I4

_I17', 'I4_I18', 'I4_I19', 'I4_I20', 'I4_I21', 'I4_I22', 'I4_I23', 'I4_I24', 'I4_I25', 'I4_Corr_P2P1_Amp', 'I4
_Corr_TTP_Amp', 'I4_A2A1', 'I4_A3A1', 'I4_A4A1', 'I4_A5A1', 'I4_A3A2', 'I4_A4A2', 'I4_A5A2', 'I4_A
4A3', 'I4_A5A3', 'I4_A5A4', 'I5_I6', 'I5_I7', 'I5_I8', 'I5_I9', 'I5_I10', 'I5_I11', 'I5_I12', 'I5_I13', 'I5_I14',
'I5_I15', 'I5_I16', 'I5_I17', 'I5_I18', 'I5_I19', 'I5_I20', 'I5_I21', 'I5_I22', 'I5_I23', 'I5_I24', 'I5_I25', 'I5_
Corr_P2P1_Amp', 'I5_Corr_TTP_Amp', 'I5_A2A1', 'I5_A3A1', 'I5_A4A1', 'I5_A5A1', 'I5_A3A2', 'I5_A
4A2', 'I5_A5A2', 'I5_A4A3', 'I5_A5A3', 'I5_A5A4', 'I6_I7', 'I6_I8', 'I6_I9', 'I6_I10', 'I6_I11', 'I6_I12', 'I6
_I13', 'I6_I14', 'I6_I15', 'I6_I16', 'I6_I17', 'I6_I18', 'I6_I19', 'I6_I20', 'I6_I21', 'I6_I22', 'I6_I23', 'I6_I24'
, 'I6_I25', 'I6_Corr_P2P1_Amp', 'I6_Corr_TTP_Amp', 'I6_A2A1', 'I6_A3A1', 'I6_A4A1', 'I6_A5A1', 'I6
_A3A2', 'I6_A4A2', 'I6_A5A2', 'I6_A4A3', 'I6_A5A3', 'I6_A5A4', 'I7_I8', 'I7_I9', 'I7_I10', 'I7_I11', 'I7_I
12', 'I7_I13', 'I7_I14', 'I7_I15', 'I7_I16', 'I7_I17', 'I7_I18', 'I7_I19', 'I7_I20', 'I7_I21', 'I7_I22', 'I7_I23', '
I7_I24', 'I7_I25', 'I7_Corr_P2P1_Amp', 'I7_Corr_TTP_Amp', 'I7_A2A1', 'I7_A3A1', 'I7_A4A1', 'I7_A5
A1', 'I7_A3A2', 'I7_A4A2', 'I7_A5A2', 'I7_A4A3', 'I7_A5A3', 'I7_A5A4', 'I8_I9', 'I8_I10', 'I8_I11', 'I8_I1
2', 'I8_I13', 'I8_I14', 'I8_I15', 'I8_I16', 'I8_I17', 'I8_I18', 'I8_I19', 'I8_I20', 'I8_I21', 'I8_I22', 'I8_I23', 'I
8_I24', 'I8_I25', 'I8_Corr_P2P1_Amp', 'I8_Corr_TTP_Amp', 'I8_A2A1', 'I8_A3A1', 'I8_A4A1', 'I8_A5A
1', 'I8_A3A2', 'I8_A4A2', 'I8_A5A2', 'I8_A4A3', 'I8_A5A3', 'I8_A5A4', 'I9_I10', 'I9_I11', 'I9_I12', 'I9_I1
3', 'I9_I14', 'I9_I15', 'I9_I16', 'I9_I17', 'I9_I18', 'I9_I19', 'I9_I20', 'I9_I21', 'I9_I22', 'I9_I23', 'I9_I24', 'I
9_I25', 'I9_Corr_P2P1_Amp', 'I9_Corr_TTP_Amp', 'I9_A2A1', 'I9_A3A1', 'I9_A4A1', 'I9_A5A1', 'I9_A
3A2', 'I9_A4A2', 'I9_A5A2', 'I9_A4A3', 'I9_A5A3', 'I9_A5A4', 'I10_I11', 'I10_I12', 'I10_I13', 'I10_I14',
'I10_I15', 'I10_I16', 'I10_I17', 'I10_I18', 'I10_I19', 'I10_I20', 'I10_I21', 'I10_I22', 'I10_I23', 'I10_I24', '
I10_I25', 'I10_Corr_P2P1_Amp', 'I10_Corr_TTP_Amp', 'I10_A2A1', 'I10_A3A1', 'I10_A4A1', 'I10_A5
A1', 'I10_A3A2', 'I10_A4A2', 'I10_A5A2', 'I10_A4A3', 'I10_A5A3', 'I10_A5A4', 'I11_I12', 'I11_I13', 'I1
1_I14', 'I11_I15', 'I11_I16', 'I11_I17', 'I11_I18', 'I11_I19', 'I11_I20', 'I11_I21', 'I11_I22', 'I11_I23', 'I1
1_I24', 'I11_I25', 'I11_Corr_P2P1_Amp', 'I11_Corr_TTP_Amp', 'I11_A2A1', 'I11_A3A1', 'I11_A4A1',
'I11_A5A1', 'I11_A3A2', 'I11_A4A2', 'I11_A5A2', 'I11_A4A3', 'I11_A5A3', 'I11_A5A4', 'I12_I13', 'I12_
I14', 'I12_I15', 'I12_I16', 'I12_I17', 'I12_I18', 'I12_I19', 'I12_I20', 'I12_I21', 'I12_I22', 'I12_I23', 'I12_I
24', 'I12_I25', 'I12_Corr_P2P1_Amp', 'I12_Corr_TTP_Amp', 'I12_A2A1', 'I12_A3A1', 'I12_A4A1', 'I1
2_A5A1', 'I12_A3A2', 'I12_A4A2', 'I12_A5A2', 'I12_A4A3', 'I12_A5A3', 'I12_A5A4', 'I13_I14', 'I13_I1
5', 'I13_I16', 'I13_I17', 'I13_I18', 'I13_I19', 'I13_I20', 'I13_I21', 'I13_I22', 'I13_I23', 'I13_I24', 'I13_I2
5', 'I13_Corr_P2P1_Amp', 'I13_Corr_TTP_Amp', 'I13_A2A1', 'I13_A3A1', 'I13_A4A1', 'I13_A5A1', 'I1
3_A3A2', 'I13_A4A2', 'I13_A5A2', 'I13_A4A3', 'I13_A5A3', 'I13_A5A4', 'I14_I15', 'I14_I16', 'I14_I17',
'I14_I18', 'I14_I19', 'I14_I20', 'I14_I21', 'I14_I22', 'I14_I23', 'I14_I24', 'I14_I25', 'I14_Corr_P2P1_Am
p', 'I14_Corr_TTP_Amp', 'I14_A2A1', 'I14_A3A1', 'I14_A4A1', 'I14_A5A1', 'I14_A3A2', 'I14_A4A2', 'I
14_A5A2', 'I14_A4A3', 'I14_A5A3', 'I14_A5A4', 'I15_I16', 'I15_I17', 'I15_I18', 'I15_I19', 'I15_I20', 'I1
5_I21', 'I15_I22', 'I15_I23', 'I15_I24', 'I15_I25', 'I15_Corr_P2P1_Amp', 'I15_Corr_TTP_Amp', 'I15_A
2A1', 'I15_A3A1', 'I15_A4A1', 'I15_A5A1', 'I15_A3A2', 'I15_A4A2', 'I15_A5A2', 'I15_A4A3', 'I15_A5A
3', 'I15_A5A4', 'I16_I17', 'I16_I18', 'I16_I19', 'I16_I20', 'I16_I21', 'I16_I22', 'I16_I23', 'I16_I24', 'I16_
I25', 'I16_Corr_P2P1_Amp', 'I16_Corr_TTP_Amp', 'I16_A2A1', 'I16_A3A1', 'I16_A4A1', 'I16_A5A1', '
I16_A3A2', 'I16_A4A2', 'I16_A5A2', 'I16_A4A3', 'I16_A5A3', 'I16_A5A4', 'I17_I18', 'I17_I19', 'I17_I2

0', 'I17_I21', 'I17_I22', 'I17_I23', 'I17_I24', 'I17_I25', 'I17_Corr_P2P1_Amp', 'I17_Corr_TTP_Amp', 'I17_A2A1', 'I17_A3A1', 'I17_A4A1', 'I17_A5A1', 'I17_A3A2', 'I17_A4A2', 'I17_A5A2', 'I17_A4A3', 'I17_A5A3', 'I17_A5A4', 'I18_I19', 'I18_I20', 'I18_I21', 'I18_I22', 'I18_I23', 'I18_I24', 'I18_I25', 'I18_Corr_P2P1_Amp', 'I18_Corr_TTP_Amp', 'I18_A2A1', 'I18_A3A1', 'I18_A4A1', 'I18_A5A1', 'I18_A3A2', 'I18_A4A2', 'I18_A5A2', 'I18_A4A3', 'I18_A5A3', 'I18_A5A4', 'I19_I20', 'I19_I21', 'I19_I22', 'I19_I23', 'I19_I24', 'I19_I25', 'I19_Corr_P2P1_Amp', 'I19_Corr_TTP_Amp', 'I19_A2A1', 'I19_A3A1', 'I19_A4A1', 'I19_A5A1', 'I19_A3A2', 'I19_A4A2', 'I19_A5A2', 'I19_A4A3', 'I19_A5A3', 'I19_A5A4', 'I20_I21', 'I20_I22', 'I20_I23', 'I20_I24', 'I20_I25', 'I20_Corr_P2P1_Amp', 'I20_Corr_TTP_Amp', 'I20_A2A1', 'I20_A3A1', 'I20_A4A1', 'I20_A5A1', 'I20_A3A2', 'I20_A4A2', 'I20_A5A2', 'I20_A4A3', 'I20_A5A3', 'I20_A5A4', 'I21_I22', 'I21_I23', 'I21_I24', 'I21_I25', 'I21_Corr_P2P1_Amp', 'I21_Corr_TTP_Amp', 'I21_A2A1', 'I21_A3A1', 'I21_A4A1', 'I21_A5A1', 'I21_A3A2', 'I21_A4A2', 'I21_A5A2', 'I21_A4A3', 'I21_A5A3', 'I21_A5A4', 'I22_I23', 'I22_I24', 'I22_I25', 'I22_Corr_P2P1_Amp', 'I22_Corr_TTP_Amp', 'I22_A2A1', 'I22_A3A1', 'I22_A4A1', 'I22_A5A1', 'I22_A3A2', 'I22_A4A2', 'I22_A5A2', 'I22_A4A3', 'I22_A5A3', 'I22_A5A4', 'I23_I24', 'I23_I25', 'I23_Corr_P2P1_Amp', 'I23_Corr_TTP_Amp', 'I23_A2A1', 'I23_A3A1', 'I23_A4A1', 'I23_A5A1', 'I23_A3A2', 'I23_A4A2', 'I23_A5A2', 'I23_A4A3', 'I23_A5A3', 'I23_A5A4', 'I24_I25', 'I24_Corr_P2P1_Amp', 'I24_Corr_TTP_Amp', 'I24_A2A1', 'I24_A3A1', 'I24_A4A1', 'I24_A5A1', 'I24_A3A2', 'I24_A4A2', 'I24_A5A2', 'I24_A4A3', 'I24_A5A3', 'I24_A5A4', 'I25_Corr_P2P1_Amp', 'I25_Corr_TTP_Amp', 'I25_A2A1', 'I25_A3A1', 'I25_A4A1', 'I25_A5A1', 'I25_A3A2', 'I25_A4A2', 'I25_A5A2', 'I25_A4A3', 'I25_A5A3', 'I25_A5A4']

A Summary of the correlation coefficients for all features included in the model is presented below. Features are presented in ascending order and are not identified due to intellectual property reasons.

**Features**	**Absolute Spearman Correlation**
1	0.07
3	0.14
4	0.15
5	0.18
8	0.18
15	0.18
2	0.22

13	0.23
11	0.24
6	0.25
7	0.26
9	0.26
10	0.26
12	0.26
14	0.27

Supplementary Table 2. Summary of the results obtained for each combination of hyperparameters, showing how they impacted model performance on the training and testing datasets. Metrics such as mean absolute error (MAE) and mean squared error (MSE) were used to evaluate each configuration. The final selection of hyperparameters was based on the combination that achieved the best balance between performance on the validation dataset and generalizability, avoiding overfitting.

**Hyperparameter Combination**	**Max iter**	**Max leaf nodes**	**Learning Rate**	**Max depth**	**Training MAE**	**Test MAE**	**Training MSE**	**Test MSE**
**1**	1000	50	0.005	50	1.51 ± 0.18	1.97 ± 0.37	2.17 ± 0.23	5.71 ± 0.46
**2**	1000	50	0.005	15	1.72 ± 0.31	2.43 ± 0.57	2.97 ± 0.73	8.51 ± 0.86
**3**	4000	50	0.005	50	0.21 ± 0.13	1.45 ± 0.24	0.33 ± 0.17	4.60 ± 0.39
**4 (Final Selection)**	4000	50	0.005	15	0.91 ± 0.01	1.23 ± 0.02	1.88 ± 0.05	3.68 ± 0.34

Iter, iterations; **MAE**, mean absolute error; **MSE**, mean squared error; max, maximum; **min**, minimum.

Supplementary Figure 1. Individual concurrent trends of invasive and noninvasive intracranial pressure methods. The charts labeled a and b below present a graphical analysis of ICP and eICP trends for each patient presenting mean absolute errors below 2 mmHg. It is noteworthy to observe instances where the lines corresponding to ICP and eICP exhibit overlap. ICP: intracranial pressure, eICP: estimated intracranial pressure.

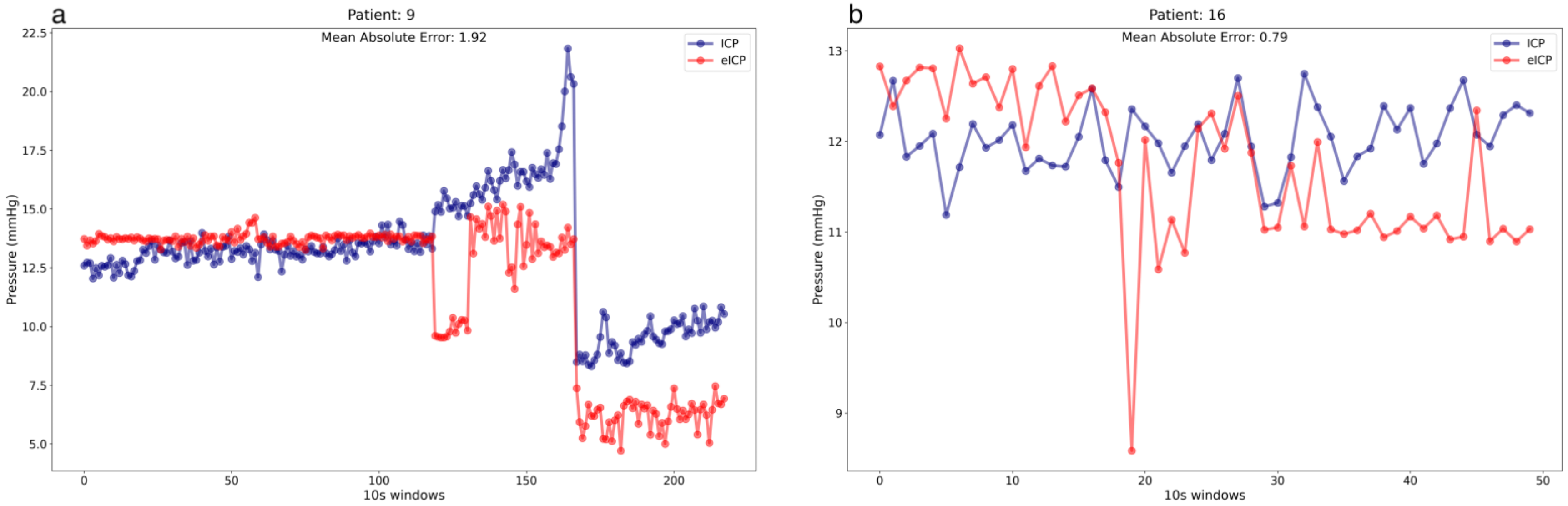

Supplementary Figure 2. Individual concurrent trends of invasive and noninvasive intracranial pressure methods. The charts labeled a to l below present a graphical analysis of ICP and eICP trends for each patient presenting mean absolute errors between 2 and 4 mmHg. It is noteworthy to observe instances where the lines corresponding to ICP and eICP exhibit overlap. ICP: intracranial pressure, eICP: estimated intracranial pressure.

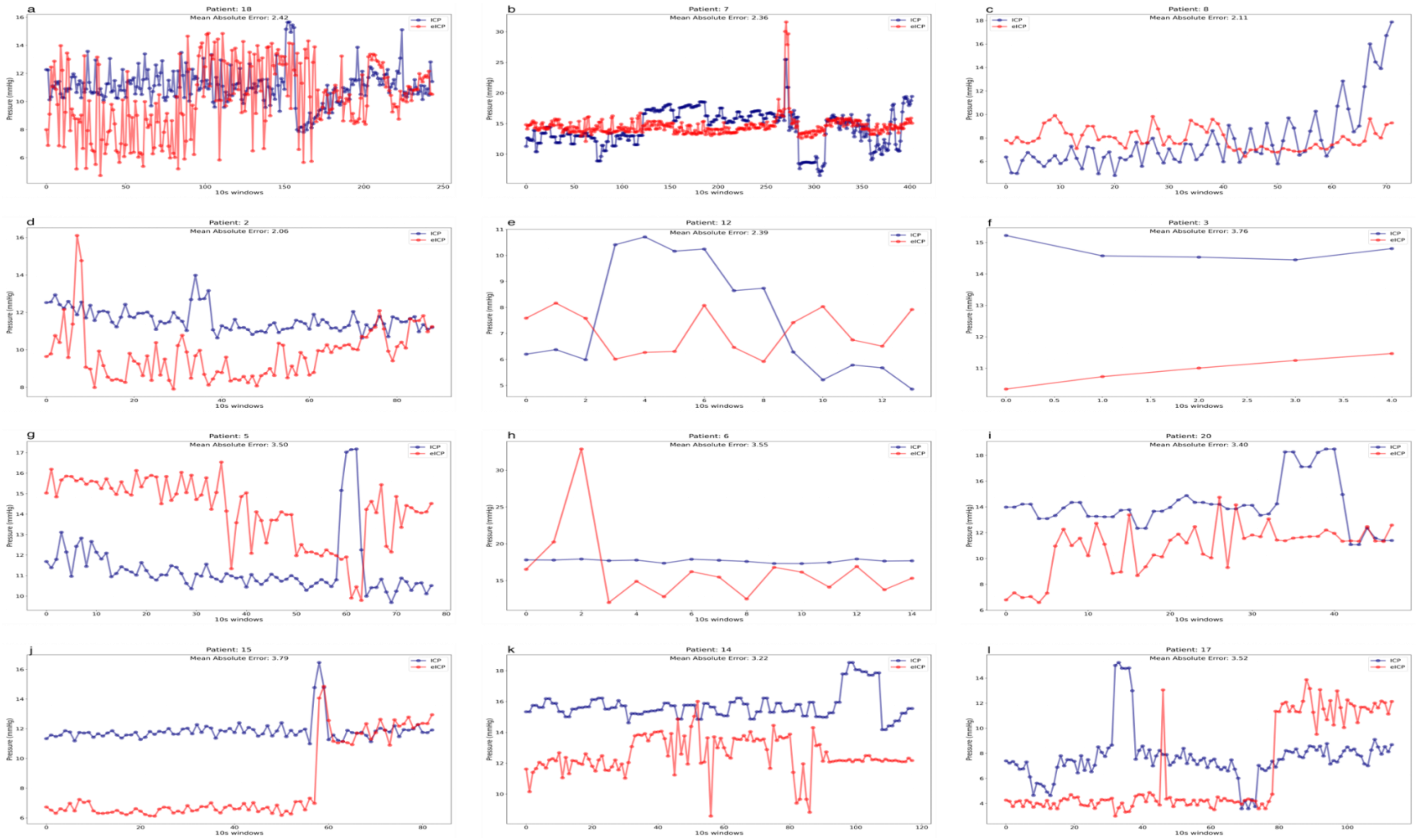

Supplementary Figure 3. Individual concurrent trends of invasive and noninvasive intracranial pressure methods. The charts labeled a to f below present a graphical analysis of ICP and eICP trends for each patient presenting mean absolute errors between 4 and 6 mmHg. It is noteworthy to observe instances where the lines corresponding to ICP and eICP exhibit overlap. ICP: intracranial pressure, eICP: estimated intracranial pressure.

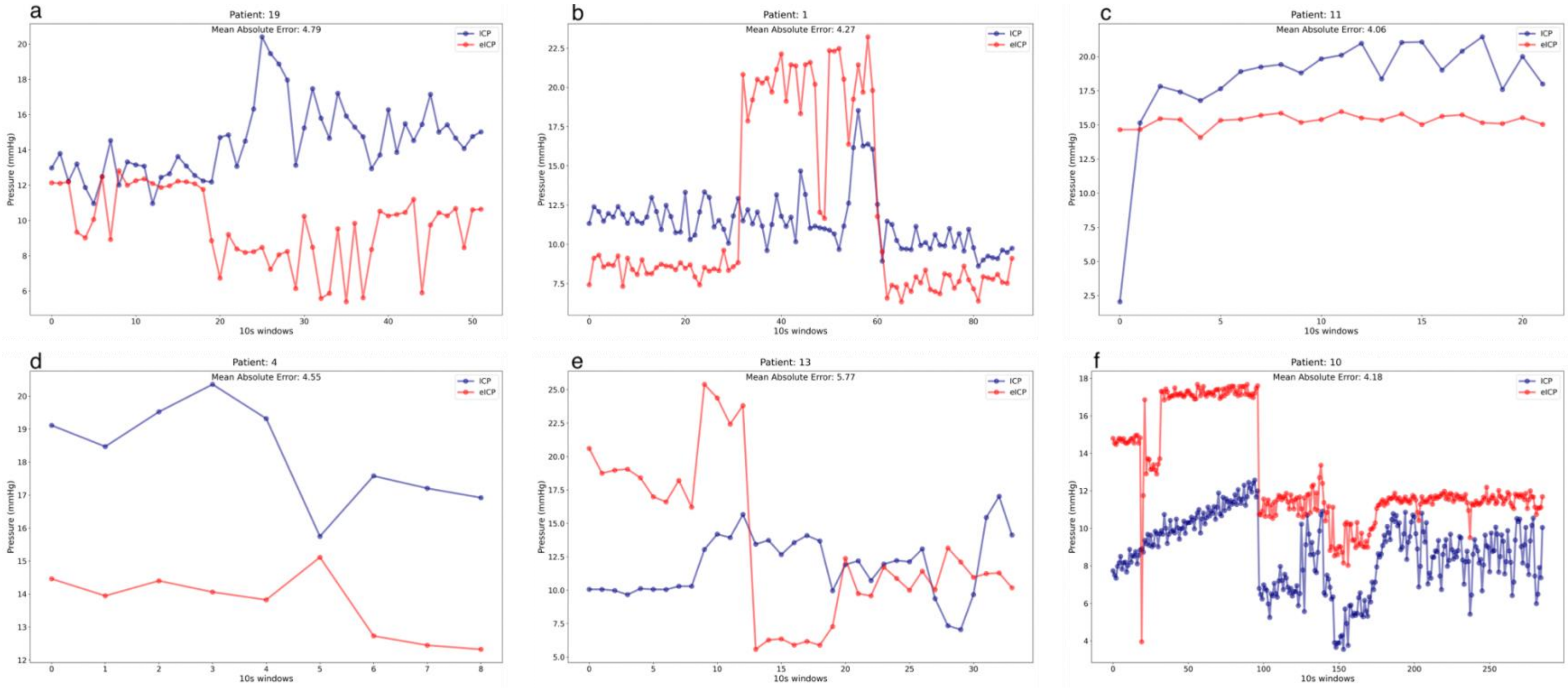

Supplement: Supplementary file 1 — Supplementary Materials [file 41746_2025_1463_MOESM1_ESM.pdf]
